# Outcomes of Implementing a Webinar-Based Strategy to Improve Spinal Cord Injury Knowledge and Community Building: Convergent Mixed Methods Study

**DOI:** 10.2196/46575

**Published:** 2023-06-23

**Authors:** Katelyn Brehon, Rob MacIsaac, Zahra Bhatia, Taryn Buck, Rebecca Charbonneau, Steven Crochetiere, Scott Donia, Jason Daoust, Chester Ho, Hardeep Kainth, Janee Loewen, Brandice Lorch, Kiesha Mastrodimos, Brittney Neunzig, Elizabeth Papathanassoglou, Rajvir Parmar, Kiran Pohar Manhas, Terry Tenove, Elysha Velji, Adalberto Loyola-Sanchez

**Affiliations:** 1 Department of Physical Therapy University of Alberta Edmonton, AB Canada; 2 Spinal Cord Injury Alberta Edmonton, AB Canada; 3 Alberta Health Services Edmonton, AB Canada; 4 Department of Clinical Neurosciences University of Calgary Calgary, AB Canada; 5 Alberta Health Services Calgary, AB Canada; 6 Praxis Spinal Cord Institute Vancouver, BC Canada; 7 Department of Medicine University of Alberta Edmonton, AB Canada; 8 Patient Partner Edmonton, AB Canada; 9 Faculty of Nursing University of Alberta Edmonton, AB Canada

**Keywords:** spinal cord injury, telehealth, webinars, mixed methods, implementation

## Abstract

**Background:**

COVID-19 disrupted services received by persons with spinal cord injury (SCI) worldwide. The International Disability Alliance declared the need for a disability-inclusive response to the COVID-19 crisis, as decreased access to health care services for individuals living with varying levels of function was unacceptable. As a result, an SCI community in Canada created a novel webinar-based strategy aimed at improving access to self-management information for people living with SCI and other stakeholders. However, although telehealth practices have previously been used effectively in SCI management and rehabilitation, little to no scholarship has investigated the outcomes of implementing a webinar-based telehealth strategy in this population.

**Objective:**

This study aims to understand the outcomes of implementing the webinar series. Specifically, the authors aimed to determine the reach of the series; understand its impact on social connectedness, perceptions of disability, and overall quality of interactions among persons with SCI, their families, service providers, and the public at large; and explore the long-term sustainability of the initiative.

**Methods:**

The authors implemented a community-based participatory strategy to define a convergent mixed methods design to triangulate qualitative and quantitative data collected simultaneously. Quantitative methods included pop-up questions administered during the live webinars, surveys administered following webinars, and an analysis of YouTube analytics. Qualitative methods included semistructured interviews with persons with SCI and health care providers who attended at least one webinar. The results were integrated, following methods adapted from Creswell and Clark.

**Results:**

A total of 234 individuals attended at least 1 of the 6 webinars that took place during the 6-month study period. In total, 13.2% (31/234) of the participants completed the postwebinar survey, and 23% (7/31) participated in the semistructured interviews. The reach of the webinar series was mainly to persons with SCI, followed by health professionals, with most of them living in urban areas. The topics *sexuality* and *research* were the most viewed on YouTube. The knowledge disseminated during the webinars was mainly perceived as valid and useful, related to the fact that the presentation format involved people with lived experience and clinical experts. The webinars did not necessarily help build a new extended community of people involved in SCI but helped strengthen the existing community of people with SCI in Alberta. The webinar positively influenced the perceptions of *normality* and *disability* regarding people with SCI. The webinar format was perceived as highly usable and accessible.

**Conclusions:**

The webinar series was associated with improved participant knowledge of what is possible to achieve after an SCI and their perceptions of disability. The long-term implementation of this initiative is feasible, but further considerations to increase its reach to rural areas and ensure the integration of diverse individuals should be taken.

## Introduction

### Background

COVID-19 disrupted services received by persons with spinal cord injury (SCI) worldwide [1]. The International Disability Alliance declared the need for a disability-inclusive response to the COVID-19 crisis, as decreased access to health care services for individuals living with varying levels of function, including those with SCI, was unacceptable [2]. As a result, the SCI community in Alberta, Canada, created a novel telehealth, webinar-based strategy aimed at improving access to self-management information for people living with SCI and other stakeholders: the “Alberta Spinal Cord Injury Community of Interactive Learning Series” (AB-SCILS). For the purposes of this study, the authors defined *telehealth* in a broader sense than the typical definition. Specifically, the authors defined *telehealth* based on the *New England Journal of Medicine* definition of *telehealth* as applied to education and patient engagement: technology used to provide health care education and allow patients to take more control of their well-being by empowering self-management and fostering spaces of emotional support [3].

Telehealth practices have previously been used in SCI management and rehabilitation and found to be effective. A systematic review (N=25) found that virtual reality–based technologies for SCI rehabilitation enhanced motor function, aerobic function, balance, and psychological aspects related to SCI while also reducing pain [3]. The authors also noted that virtual reality–based technologies were highly motivating and engaging environments for rehabilitation [4]. A pretest-posttest study of the effectiveness of a home-based telerehabilitation (ie, web-based delivery of rehabilitation) program on reducing shoulder pain and improving shoulder function (N=16) in people with SCI found that pain was reduced, whereas function was improved [5]. Telehealth modalities have also been found to be favored for the self-management of persons with SCI as they mitigate the transportation and mobility barriers of in-person appointments [6]. A recent community-engaged pilot study evaluated the usability and effectiveness of a telehealth self-management intervention for persons with SCI (N=10) [6]. The pilot included the creation and viewing of instructional videos [7]. Participants were satisfied with the initiative and found the videos motivating and relatable owing to those in the videos being people with lived experience and to the videos being acceptable lengths [7].

Previous research finding that webinars can substantially improve the self-management of people living with chronic conditions (such as cancer [8]) and are effective in promoting adult learning [9] was used as the justification to develop the AB-SCILS. However, little to no scholarship has investigated the outcomes of implementing a webinar-based telehealth strategy among individuals with SCI. With a forced shift to telehealth because of the onset of the pandemic and its continued use in the present day, research into the outcomes of implementing a strategy such as the AB-SCILS is warranted.

### Organizational Context

The AB-SCILS was developed by a group of stakeholders, representing (1) the provincial health care system (ie, Alberta Health Services); (2) Spinal Cord Injury Alberta, the main community organization supporting persons with SCI in the province; and (3) Praxis, a Canadian-based not-for-profit organization that leads global collaboration in SCI research, innovation, and care. The AB-SCILS was developed in response to the need for a disability-inclusive response to the COVID-19 pandemic for individuals living with SCI. The webinars were co-designed by persons with SCI (ie, lived experience experts) and clinical experts so that both firsthand and clinical perspectives were shared during each webinar. All webinars were recorded and posted on a dedicated YouTube channel and are open to the public.

Through consultation with an advisory panel of community partners living with SCI (discussed in the following sections), the authors anticipated that the AB-SCILS would increase the audience’s knowledge of SCI, thus affecting their perceptions of SCI-related disability, as well as improve the ability of persons with SCI to self-manage by building a sense of community, thus ameliorating the isolating effects of the COVID-19 pandemic.

### Research Aims

This study aimed to understand the outcomes of implementing the AB-SCILS. Specifically, the authors aimed to (1) determine the reach of the AB-SCILS to the various members of the SCI community in Alberta (ie, persons with SCI; their families; service providers; and the broader community, including rural communities where support is limited); (2) understand the impact of the initiative on social connectedness, perceptions of disability, and overall quality of interactions among persons with SCI, their families, service providers, and the public at large; and (3) explore the long-term sustainability of the AB-SCILS.

## Methods

### Overview

This was a mixed methods study, and the methods are described in detail in a published study protocol [10]. Methods included pop-up questions administered during live webinars, surveys, semistructured interviews, and analysis of YouTube analytics. In summary, pop-up questions and YouTube analytics were used to describe the population attending the webinars as well as those who accessed them via YouTube after the live sessions, thereby providing information on the initiative’s reach. Surveys and semistructured interviews were used to understand the impact of the AB-SCILS on community building and perceptions of disability. The authors aimed to understand both the reach and impact to provide insights on the sustainability of the initiative. If the AB-SCILS reached the intended audience and had the desired impact, the authors perceived that it should be sustainable.

### Study Co-design

The authors used a community-based participatory strategy to define a convergent mixed methods design [11]. At the beginning of the evaluation process, the authors established an advisory panel of 3 individuals with SCI to understand how they thought the AB-SCILS should be evaluated. At the end of the initial meeting, the authors inquired into these individuals’ preferred level of participation in the study using the International Association for Public Participation 2 Spectrum of Public Participation [12] to ground the discussion. The advisory panel chose to be *involved*, thus working directly with the research team throughout the process to ensure that their concerns with and visions for the AB-SCILS were understood and considered. As a result, the authors hosted bimonthly discussions with the panel to garner feedback on the ongoing analysis and preliminary findings, including suggestions on how results should be interpreted.

**Figure 1:**
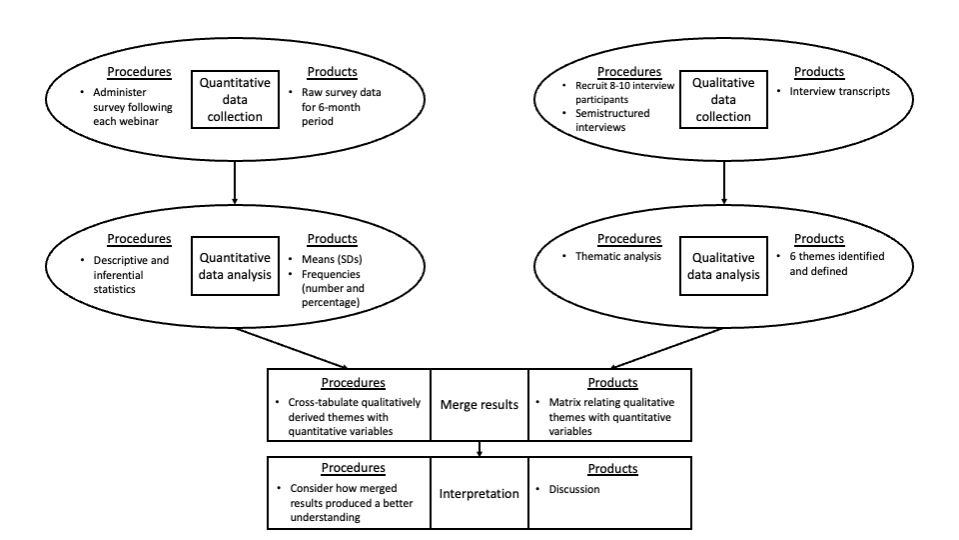
Flowchart depicting procedures and products in convergent mixed methods design (adapted from Creswell and Clark [11]).

The authors used a convergent mixed methods design [11] to triangulate qualitative and quantitative data collected simultaneously. The findings from each data source were subsequently compared to obtain a more complete understanding of the AB-SCILS' impact (Figure 1). A convergent mixed methods design brings together the strengths and weaknesses of both qualitative and quantitative methods [11]. This design is oriented toward real-world practice, with a focus on the importance of the research question being investigated rather than on the methodologies used [11]. Qualitative methods included semistructured interviews with persons with SCI and health care providers who attended at least one of the AB-SCILS webinars. Quantitative methods included surveys administered following AB-SCILS webinars and an analysis of YouTube analytics.

The authors used Sandelowski’s framework [13] for qualitative description to inform the qualitative component of the study. In qualitative description, a researcher does not deliberately choose to describe an event in terms of a specific framework or system but rather presents the facts of the study in layman’s terms [13]. Qualitative description studies often draw their results from naturalistic inquiry or the study of something in its natural state [13].

### Study Population

#### Overview

In consultation with the advisory panel, the authors determined that the AB-SCILS should not be solely targeted toward people with SCI. To build a broader SCI community, it was determined that the AB-SCILS should target persons with SCI (including patients who were hospitalized) and their families, health care professionals that are a part of their care journey (eg, general practitioners, nurses, rehabilitation providers [physiotherapists and occupational therapists], social workers, and physiatrists), and members of the broader community (eg, teachers and city designers). Therefore, the population of interest included all individuals who attended the AB-SCILS between November 2020 and April 2021.

#### Inclusion and Exclusion Criteria

Participants had to be aged ≥18 years and have attended at least one AB-SCILS webinar. They also had to be able to read and understand English on their own or have support (ie, language translation) from their family or friends. There were no exclusion criteria applied in this study.

### Recruitment and Sampling

The authors sought a convenience sample of approximately 30% of all AB-SCILS attendees for the pop-up questions and follow-up surveys. The link to the follow-up survey was sent to all webinar attendees via email. All attendees had to provide an email address when registering for each webinar. Attendees were welcome to fill out the surveys more than once during the 6-month evaluation period and were encouraged to do so by having their names be entered into a draw for a tablet at the end of the project.

The authors aimed to recruit approximately 8 to 10 AB-SCILS attendees to participate in the interviews. Individuals were asked whether they consented to be contacted for a follow-up interview after completing the survey. If attendees consented to be contacted, their contact information was shared with the study coordinator. The study coordinator then contacted these attendees to go through the informed consent process and arrange a date and time for the phone interview.

### Data Collection

To understand the reach of the AB-SCILS, the authors sought descriptive statistics of those attending the webinars within the study period as well as those who accessed the webinars on YouTube following the live sessions. All individuals who attended the webinars live had the chance to complete a series of pop-up questions during each webinar. Pop-up questions were administered during each webinar held within the study period. Questions included (1) type of participant (ie, person with SCI, family member of someone with SCI, health care provider, researcher, program manager, student, or other); (2) whether attendees were from Edmonton, Calgary, elsewhere in Alberta, or outside Alberta; (3) attendance record (ie, first webinar, attended 1-3 webinars, attended 4-6 webinars, or attended >6 webinars); and (4) whether they had accessed past webinars on YouTube. To understand the reach following the live session, aggregate YouTube analytics data were accessed through the AB-SCILS YouTube account. YouTube data included total views, number of unique viewers, shares, comments added, and watch time (hours) per webinar.

To measure the impact and sustainability of the AB-SCILS, the authors completed follow-up surveys and semistructured interviews. Follow-up surveys were sent following each webinar to every attendee. Survey responses were captured using REDCap (Research Electronic Data Capture; Vanderbilt University) [14]. The survey package was codeveloped with the advisory panel and included questions about telehealth usability as well as the ability of the AB-SCILS to foster social connectedness and change perceptions of disability. Questions related to social connectedness and telehealth usability were adapted from the Sense of Virtual Community questionnaire [15] and the Telehealth Usability Questionnaire [16], respectively. Questions regarding challenging perceptions of living with a disability were written in consultation with the advisory panel. A more detailed explanation of each survey can be found in Textbox 1, and all the questions can be found in the *Results* section. The survey package also included demographic questions (eg, age, sex, educational background, and occupational status).

**Alberta Spinal Cord Injury Community of Interactive Learning Series (AB-SCILS) evaluation survey details and scoring instructions.**

**Sense of Virtual Community questionnaire (SOVC)**

- The authors adapted the SOVC questionnaire by Abfalter et al [15], which has 15 items and is measured on a Likert scale from 0 to 3, with 0 indicating “not at all” and 3 indicating “completely.” The scale measures facets of membership in a web-based community, influence on the community, integration and fulfillment of needs, and shared emotional connection among members [15].

**Telehealth Usability Questionnaire (TUQ)**

- The TUQ-10 measures the following domains: usefulness, ease of use and learnability, interface quality, reliability, and satisfaction and future use [16]. Each domain is measured on a Likert scale from 1 to 5, with 1 indicating “strongly disagree” and 5 indicating “strongly agree” [16].

**Challenging perceptions of disability questionnaire**

- The challenging perceptions questionnaire contained 11 questions. In total, 5 questions were measured on a 5-point Likert scale ranging from “strongly disagree” to “strongly agree” and analyzed perceived levels of knowledge before and after each webinar, as well as perceptions of whether individuals with lived experience of spinal cord injury (SCI) could lead normal, meaningful, and independent lives. The remaining 6 questions were measured on a 7-point Likert scale ranging from “always” to “never” and analyzed feelings people had when interacting with people with lived experience of SCI (eg, sadness, happiness, anger, anxiety, calmness, and feeling sorry).

The interviews were conducted by an experienced interviewer trained in qualitative methods. The semistructured interviews were 1:1 and remote (conducted by phone). The interview guide was informed by the advisory panel. The interviews asked about webinar experiences, perceived successes and challenges of the webinars themselves, takeaways of the webinars (ie, whether their views on SCI were challenged and how so), and the ability of the AB-SCILS to build a broader SCI community. The interviews also asked about social inclusion, quality of life, and perceptions of living with a disability. Probing questions were used to elicit greater description as necessary. The interviews were recorded using a digital audio recorder and were confidentially transcribed verbatim.

### Data Analysis

#### Quantitative Analysis

Pop-up and YouTube analytics data were analyzed using Microsoft Excel (Microsoft Corp). The authors calculated the means and SDs of any interval data and the frequencies (number and percentage) of any categorical data. Survey data were analyzed cross-sectionally and longitudinally using SPSS (IBM Corp) and Stata (StataCorp) [17,18], and 2 members of the team completed the analyses separately.

#### Qualitative Analysis

Thematic analysis was completed on all interview transcripts using a specialized software for qualitative data management (Dedoose; SocioCultural Research Consultants) [19]. Thematic analysis involved reading through the transcripts and grouping similar ideas together as codes. Codes were then grouped together into overarching themes. A member of the research team (KB) coded all the transcripts. To ensure accuracy of coding, 2 other members of the research team (RM and AL-S) coded a portion of the transcripts. In total, 3 research team members (KB, RM, and AL-S) met to discuss codes and themes and develop a common analytical coding framework. The transcripts were then reread to ensure that they were coded appropriately based on the agreed-upon analytical coding framework.

The 3 members involved in the analysis were a Master’s of Public Health student with no previous experience conducting research with the SCI community (KB), a social worker with SCI lived experience and a long trajectory supporting people with SCI in the community (RM), and an academic physiatrist with clinical experience in SCI and a doctorate in rehabilitation sciences (AL-S). These members continuously reflected on their assumptions while analyzing the data, and the 3 agreed on the importance of challenging preconceived notions of *normality* (ie, what it meant to lead a *normal* life as someone with SCI), as these often result in unfair service access for people with disabilities. Rigor was promoted through an audit trail of decisions for accountability, open-ended questions to prioritize participant voices, the use of a thick description of the context in which the AB-SCILS operates, collaborative coding for discussion of subjectivity and openness in analysis, and reflexive journaling and discussion [20].

#### Integrated Analysis

To triangulate findings from the quantitative and qualitative data arms [11], the authors began with each of the key themes generated from the qualitative analyses. A key statement was then written that embodied what each theme was about. The authors then discussed which questions from the follow-up survey addressed the factors discussed in each theme. Next, specific assumptions were tested based on each relevant follow-up survey question using ordinal directions expressed by frequency of level of agreement, coded dichotomously. If assumptions were confirmed, meaning that ≥80% of the responses were in whatever ordinal direction was expected (ie, ≥80% agreement or disagreement), the authors completed no further assumption testing. If assumptions were not confirmed, inferential tests were conducted to explore the effects of potential predictive variables (type of participant, sex, and educational level) and their association with responses of agreement or disagreement. Specifically, the authors estimated odds ratios (ORs) through simple logistic regression modeling considering a Cronbach α value of .05 as significant.

### Ethics Approval, Informed Consent, and Participant Reimbursement

The University of Alberta Health Research Ethics Board approved this study (Pro00102178). All participants provided informed consent before taking part in the study. All study data were deidentified, and a pseudonym was assigned to participant contributions where appropriate. All participants who completed the survey were entered into a draw for a tablet computer (value of CAD $200 [US $148.06]). Those who participated in an interview received a CAD $20 (US $14.81) gift card.

## Results

### AB-SCILS Reach

There were 234 individuals who attended at least 1 of the 6 webinars that occurred during the 6-month study period (ie, November 2020 to April 2021). Not all attendees answered the pop-up questions, leading to variations in the number of total responses for each question. The results from the pop-up questions (Table 1) revealed that most webinar participants were persons with SCI (66/147, 44.9%) or health care providers (41/147, 27.9%), had attended >6 webinars (42/117, 35.9%), and were from either the Edmonton (68/140, 48.6%) or Calgary (50/140, 35.7%) areas. There was an almost even split between whether participants had viewed a webinar on YouTube (58/117, 49.6%) or not (59/117, 50.4%) following the live session.

YouTube analytics results are presented in Table 2. The webinar with the most YouTube views was “Episode 15—Sexuality After SCI” (299/771, 38.7%). This webinar also had the highest number of unique viewers (212/594, 35.7%) and shares (6/22, 27%) and the longest watch time (22.1 hours) during the study period. “Episode 16—SCI Research and Experimental Technologies” received the most comments (4/9, 44%) on YouTube during the study period.

**Table 1:** Understanding during-webinar reach via pop-up questions.

Variable		Participants, n (%)
**Type of participant, (n=147)**		
	Person with lived experience	66 (44.9)
	Family member or caregiver	6 (4.1)
	Health care provider	41 (27.9)
	Researcher	7 (4.8)
	Program manager	13 (8.8)
	Student	4 (2.7)
	Other	10 (6.8)
**Place of residence, (n=140)**		
	Edmonton	68 (48.6)
	Calgary	50 (35.7)
	Elsewhere in Alberta	20 (14.3)
	Outside Alberta	2 (1.4)
**Webinar attendance, (n=117)**		
	First webinar	31 (26.5)
	Attended 1-3 webinars	20 (17.1)
	Attended 4-6 webinars	24 (20.5)
	Attended >6 webinars	42 (35.9)
**Accessed webinars on YouTube, (n=117)**		
	Yes	58 (49.6)
	No	59 (50.4)

**Table 2:** Understanding postwebinar reach via YouTube analytics.

	Total views, (n=772), n (%)	Unique viewers, (n=594), n (%)	Shares, (n=22), n (%)	Comments, (n=9), n (%)	Watch time, hours
Episode 12—A Conversation With Synaptic Neuro Rehabilitation and Reyu Recovery Centre	71 (9.2)	53 (8.9)	5 (22.7)	0 (0)	4.2
Episode 13—Activity Based Lifestyle: Ways of Staying Fit in Your Community	105 (13.6)	26 (4.4)	2 (9.1)	2 (22.2)	15.5
Episode 14—Mental Health: Depression and Coping Skills	89 (11.5)	61 (10.3)	4 (18.2)	2 (22.2)	4.4
Episode 15—Sexuality After SCI^a^	299 (38.7)	212 (35.7)	6 (27.3)	1 (11.1)	22.1
Episode 16—SCI Research and Experimental Technologies	45 (5.8)	64 (10.8)	3 (13.6)	4 (44.4)	3.7
Episode 17—Returning to Your Rural Community	163 (21.1)	178 (30)	2 (9.1)	0 (0)	17.5
Value, mean (SD)	128.7 (92.4)	99 (76.3)	3.7 (1.6)	1.5 (1.5)	11.2 (8.1)

^a^SCI: spinal cord injury.

### AB-SCILS Impact and Sustainability

#### Overview

There were 31 unique webinar attendees who completed the survey. Survey sample demographics are shown in Table 3. Most respondents were female (21/31, 68%), persons with SCI (19/31, 61%), legally married (and not separated; 16/31, 52%), White (26/31, 84%), had a bachelor’s degree (10/31, 32%), and worked 1 to 39 hours per week (14/31, 45%). A total of 19% (6/31) of the attendees completed the survey on more than one occasion. Their longitudinal data were analyzed but not reported because of the small sample size. Survey questions (with associated question numbers), which survey each question was adapted from, and the survey results are shown in Multimedia Appendix 1.

A total of 3% (7/234) of the webinar attendees participated in the interviews. Of these 7 attendees, 5 (71%) were persons with SCI and 2 (29%) were health care providers involved in SCI care. Six key themes were generated during the thematic analysis: (1) legitimacy of knowledge, (2) applying knowledge, (3) building community, (4) challenging normality, (5) meeting community needs, and (6) webinar platform usability. The authors constructed key statements directly from each theme (Table 4).

**Table 3:** Follow-up survey demographics (n=31).

Variable		Values
Age (years), mean (SD)		45 (12.8)
**Sex, n (%)**		
	Male	10 (32)
	Female	21 (68)
**Type of participant, n (%)**		
	Person with lived experience	19 (61)
	Family member	3 (10)
	Health care provider	6 (19)
	Student	1 (3)
	Manager	1 (3)
	Other	1 (3)
**Marital status, n (%)**		
	Single	6 (19)
	Legally married (not separated)	16 (52)
	Common law	6 (19)
	Separated	1 (3)
	Never legally married	0 (0)
	Divorced	1 (3)
	Prefer not to disclose	1 (3)
**Race or ethnicity, n (%)**		
	Ethnic minority	4 (13)
	White	26 (84)
	Prefer not to disclose	1 (3)
**Educational level, n (%)**		
	High school diploma or equivalent	2 (6)
	Some postsecondary education, no degree	6 (19)
	Apprenticeship	6 (19)
	Associate degree	3 (10)
	Bachelor’s degree	10 (32)
	Graduate degree	4 (13)
**Employment status, n (%)**		
	Not employed and not looking	6 (19)
	Unable to work	1 (3)
	Retired	2 (6)
	Working 1-39 hours per week	14 (45)
	Working ≥40 hours per week	4 (13)
	Prefer not to disclose	4 (13)

**Table 4:** Themes identified from the interviews with corresponding constructed key statements and exemplary quotes.

Theme and key statement		Exemplary quote
**Legitimacy of knowledge**		
	The integration of clinical and lived experience perspectives created legitimate, accessible, and approachable new knowledge.	“I guess now I’m at the point where the equal like peer and provider information is great and working together to see that it’s the two worlds working together, the two fields working together... to give a total picture is great.” [Female; person with lived experience of SCI^a^]
**Applying knowledge**		
	The AB-SCILS^b^ provided knowledge that people can apply as well as a space for people with lived experience of SCI and health care providers to apply and disseminate knowledge (ie, double knowledge translation).	“In my personal life I have a brother who is going through a process of transferring to a wheelchair so it’s also good for my personal life to know and like learn about how to help him as well as I work with families and some of these families have kids with or part of the family has physical disabilities so I definitely think that it’s worthwhile and useful.” [Female; provider]
**Building community**		
	Community was built through connections around common knowledge and empowerment of the preexisting SCI community.	“...there was a new injury there as well and she was a very shy gal and uh she didn’t say too much but she did take my number and accept that I could contact her after the meeting so that’s a very big positive coming from your meeting. That’s probably the biggest positive gold star that you could have is a new injury that was shy and kind of isolated to be able to come forward and accept new information and more contact. That’s probably the brightest star in your whole webinar right there. Is giving someone the opportunity to hope for something better.” [Male; provider and person with lived experience of SCI]
	The AB-SCILS needs to ensure that people feel like they are part of a safe space so that they can express their views and perspectives in the community.	“I felt like it was very nice to have a discussion with different people who either live with spinal cord injury or work with spinal cord injury...but...like I said did feel like it was little bit anti[workplace]. So that didn’t make me feel like I was part of that community.” [Female; provider]
	The AB-SCILS also needs to ensure that it does not exclude people by not considering the diversity and format of the webinars.	“With little kids...it makes it harder for me to get out and join those things like unless it’s okay for me to bring the kids along kind of thing...I like the zoom, I really like the zoom...for the information the online format is great...it would be a little bit more accessible for me to do online.” [Female; person with lived experience of SCI]
**Challenging normality**		
	The idea of what a “normal” life with SCI looked like was challenged through reconstruction and coconstruction of *normality* by people with lived experience of SCI and other attendees.	“...it’s a cultural shift or societal shift to...look at a person with a spinal cord injury, see their struggles and their setbacks but not look down on them in a way. Like to empathize instead of sympathize.” [Female; person with lived experience of SCI]
**Meeting community needs**		
	The purpose of the AB-SCILS was not clear to every attendee, which meant that it did not always meet the needs of all community members.	“...what exactly is the purpose of the...SCILS...like is it mainly for education?” [Female; provider]
**Webinar platform usability**		
	Connectivity, professionalism, and knowledge shared from both people with lived experience of SCI and clinicians equated to attendees viewing the webinar as usable.	“...I appreciate all of it honestly. I love that there’s the professionals there giving some of the...well-researched information because that’s always very helpful to check in with that stuff...and then having the peers speaking and hearing their experiences, very valuable, and then I think there was a break out room...for that one and that was great too because then we all got to speak about our personal experiences and connect so I appreciate all of those aspects.” [Female; person with lived experience of SCI]

^a^SCI: spinal cord injury.

^b^AB-SCILS: Alberta Spinal Cord Injury Community of Interactive Learning Series.

#### Mixed Integrated Findings

The following is a description of the integrated results organized by the categories constructed through the mixed analysis (Multimedia Appendix 2).

##### Category 1: Knowledge Disseminated During the Webinars and Its Applicability

The knowledge disseminated through the 6 webinars evaluated was perceived as valid and effective in increasing knowledge about the topics presented, which demonstrated that the pedagogical approach taken in the webinar was effective in increasing knowledge in the audience. Most survey participants (28/31, 90%) agreed that they trusted the people delivering the content and the people attending and organizing the webinar (ie, quantitative results), which aligns with the “Legitimacy of Knowledge” theme (ie, qualitative results). In addition, about half (15/31, 48%) of the survey participants reported not knowing much about the topic presented before attending, and most (26/31, 83%) agreed that their knowledge about the topic increased after participating in the webinar (ie, quantitative results).

The applicability of the knowledge disseminated during the 6 webinars evaluated was perceived as high because of its dissemination format, which included a combination of clinical and lived experience experts. The theme identified as “Applying Knowledge” demonstrated that the AB-SCILS provided a space for people with lived experience and people with professional expertise to converge and present information that was both factual and practical for daily life (ie, qualitative results). In addition, most survey participants (27/31, 87%) perceived that their needs, priorities, and goals were reflected in the webinar’s content (ie, quantitative results).

##### Category 2: AB-SCILS Impact on Community Building and Social Connectedness

The AB-SCILS did not have a strong impact on community building between people with lived experience, family members, service providers, and the public at large. However, the webinars did contribute to strengthening preexisting community connections among persons with SCI. The theme “Building Community” demonstrated that persons with SCI perceived the webinars as a platform to connect and strengthen relationships with peers (ie, qualitative results). However, narratives from participants without lived experience did not express a sense of community building with other participants when attending the webinars (ie, qualitative results). This finding aligns with the survey results, which demonstrated that, even though participants perceived webinar members as highly trustworthy, as good leaders, and as caring, they did not feel that they could talk to other participants about their problems (ie, quantitative results). In addition, survey participants did not agree with being known by other webinar participants, enjoying being with other webinar participants, or the importance of fitting in within the webinar community (ie, quantitative results). Interestingly, the authors found a significant association between reported gender and enjoying the company of other webinar participants. Men were 4.99 times (*P*=.04) more likely to report being with other members of AB-SCILS and enjoying their company in comparison with women (ie, quantitative results).

The theme “Meeting Community Needs” indicated that the purpose of the AB-SCILS as a community-building effort was not clear to all participants, and consequently, some participants’ narratives expressed that their expectations and needs were not always met when attending the webinars (ie, qualitative results). The survey findings confirmed that participants did not unanimously agree that they had important needs met when participating in the AB-SCILS (ie, quantitative results). Furthermore, subgroup inferential analyses showed that most of the participants who self-identified as health care providers (5/6, 83%) disagreed with having important needs met after participating in the webinars (OR 0.011; *P*=.06; ie, quantitative results).

##### Category 3: AB-SCILS Impact on SCI Perceptions of Normality and Disability

The webinar had an impact on the perception of whether persons with SCI can lead meaningful, “normal” lives. The theme “Challenging Normality” showed that participants were able to reconstruct the idea of what a “normal life” for a person with SCI looks like after attending the webinars (ie, qualitative results). This theme stemmed directly from the fact that the webinar content was cocreated with persons with SCI, which provided firsthand experience to participants of how successful and “normal” a person with SCI’s life could be. Survey responses supported these results, showing that most participants agreed with the statement that people with SCI can lead meaningful (31/31, 100%), normal (28/31, 90%), and independent (30/31, 97%) lives (ie, quantitative results). Interestingly, when it came to exploring emotions related to SCI perception, not all participants demonstrated positive or neutral feelings when exposed to people with SCI. Many participants disagreed with feeling *sorry* (19/31, 61%) or *sad* (18/31, 59%) when they saw a person with SCI (ie, quantitative results). Similarly, many participants agreed with feeling *happy* (13/31, 41%) or *calm* (25/31, 81%) when encountering people with SCI (ie, quantitative results). A subgroup inferential analysis showed that having a postsecondary education was associated with higher odds of feeling “happy” or “calm” when seeing a person with SCI in comparison with participants without a postsecondary education (OR 12.6; *P*=.03 and OR 11; *P*=.01, respectively; ie, quantitative results).

##### Category 4: AB-SCILS Usability

The webinar platform was perceived as highly usable and accessible. The theme “Webinar Platform Usability” suggested that the webinar platform, including its live and recorded sessions, was perceived as highly useful as it enhanced connectivity among persons with SCI in an accessible, electronic environment (ie, qualitative results). It was also perceived that the webinar usability was enhanced by the professionalism of the presenters as well as the way the content was presented, which always included peer and professional knowledge integrated in a practical way (ie, qualitative results). In addition, survey results demonstrated that the webinar platform was highly accessible, simple to use, and easy to learn, and most participants (26/31, 83%) felt that they could use it productively in a timely manner (ie, quantitative results).

## Discussion

### Principal Findings

This study showed that the reach of the AB-SCILS webinar was mainly to persons with SCI, followed by health professionals, with most of them living in urban areas. The topics *sexuality* and *research* were the most viewed afterward on YouTube. The knowledge disseminated during the webinars was mainly perceived as valid and useful, mainly because of its presentation format involving people with lived experience and clinical experts. The AB-SCILS did not necessarily help build a new extended community of people involved in SCI but helped strengthen the existing community of people with SCI in Alberta. The webinar influenced the perceptions of *normality* and disability regarding people with SCI, showing that, after attending the AB-SCILS, people agreed more with the fact that having an SCI does not preclude individuals from leading meaningful lives. Finally, the results demonstrated that the webinar format is highly usable and accessible, implying that AB-SCILS sustainability in the long term is feasible.

Most participants in the webinar were persons with SCI or health care providers (107/147, 72.8%) from either the Edmonton or Calgary area (118/140, 84.3%). This lack of rural participation may have been because it was anecdotally more challenging to get information about the AB-SCILS out to rural areas. There are often fewer individuals with SCI living rurally as well. There may also be some challenges related to limited access to technology. A study conducted in 2014 found that rural-dwelling Canadians had lower levels of internet access, with a lower number having a desktop, laptop, or mobile device with internet access at home compared with those living in urban areas [21]. The impact of this lack of internet access in rural Canada may have been amplified during the COVID-19 pandemic, with society transitioning to mainly web-based modes of communication [22]. Consequently, exploring access to web-based technology in rural areas of Alberta will be essential to improve the reach of webinar initiatives such as the AB-SCILS.

Although there was good participation in the live webinars, with >200 attendees during the study period, there were also a substantial number of views on YouTube after the live sessions. This suggests that the timing of the webinars may be a determinant of whether some participants can attend the live sessions, speaking to the importance of having the webinars available for later views. Chiswell et al [8] evaluated a suite of webinars in terms of overall experience, viewer satisfaction, self-reported changes in knowledge, and confidence to discuss webinar topics. The authors found that the main reason why individuals did not attend the live webinar was due to prior commitments, the time of day at which the webinar was scheduled, or preference to listen to the webinar at a later time [8]. Consequently, it is important to record webinars such as the AB-SCILS to improve their reach and accessibility. Recording webinars and making them available on the web will allow people to view them even if they are unable to attend live or if they want to watch the session again to reinforce their knowledge.

The webinars posted on the YouTube channel that were evaluated in this study also varied in terms of number of views, comments, shares, and watch time. The webinar with the most views and shares and the longest watch time was “Episode 15—Sexuality After SCI.” Attendees may have viewed, shared, and watched this episode the most because of perceived importance or curiosity about the topic. Previous research investigating the characteristics that drive virality, or sharing, of web-based advertisements found that information-focused content is less likely to be shared unless the information is novel or interesting in nature [23]. Furthermore, content evoking discrete positive emotions such as inspiration, warmth, amusement, and excitement was found to be more likely to be shared [23]. Although these findings were not studied in the context of webinars, it can be purported that “Episode 15—Sexuality After SCI” may have been viewed, shared, and watched more than the other AB-SCILS episodes because of viewer interest and lack of previous knowledge about the topic. This episode may have also evoked positive emotions in viewers, adding to its likelihood of being shared via social media. It will be important to further investigate the types of emotions evoked by the different content included in the AB-SCILS webinars.

The AB-SCILS worked by disseminating knowledge that was perceived as trustworthy in a format that allowed attendees to know more about the topics presented after the webinar. Previous research has demonstrated that webinars are an effective modality to improve the knowledge of viewers [24-26]. A meta-analysis (N=12 articles) found that participants developed more knowledge and skills during longer webinars compared with shorter webinars [24]. Furthermore, the authors noted differences in participant knowledge gains resulting from what webinar platform was used (ie, Cisco Webex was associated with greater knowledge gains compared with Adobe Connect [24]), which suggests that the webinar platform used may affect the perceived quality of the information being shared. Interestingly, repeating the same topic in multiple webinars did not result in greater knowledge gains compared with sharing the topic in only one webinar [24]. This suggests that, in the AB-SCILS, topics should likely only be presented once unless there is novel information to share.

The knowledge disseminated in the AB-SCILS was applicable to the needs, goals, and priorities of persons with SCI and helped increase awareness that it is possible to lead a meaningful life after SCI. However, some attendees (13/31, 42%) did not agree with being able to discuss their own problems with the webinar community, suggesting that there may be factors hindering the sharing and applicability of the knowledge presented in the webinars. A qualitative study demonstrated that having peer coaches positively affects the self-management of people with SCI [27]. In addition, a scoping review on peer-led interventions for people with SCI demonstrated that the positive effect of having peers guiding and presenting information on self-management is effective, mostly in a one-to-one format [28]. Consequently, this study’s findings may suggest that, even though the knowledge disseminated in the AB-SCILS was highly applicable (because of the participation of persons with SCI) and respected (because of the participation of clinical experts), the lack of a one-on-one space to make this knowledge more personalized hindered the potential of translating the disseminated knowledge into meaningful life changes. In other words, it is fair to say that the format of the AB-SCILS helped people change their perceptions of what is possible related to living with SCI, but its format did not allow individuals to effectively channel these new perceptions into concrete actions to improve the lives of people with SCI.

This study’s results suggest that the AB-SCILS community was built through connections regarding common knowledge and empowerment of a preexisting SCI community in the province. However, the authors identified that the AB-SCILS needs to ensure that people feel like they are part of a safe space so that they can express their views and perspectives in a community that goes beyond people with SCI and includes family members, care providers, and the public at large, ensuring that it includes people by considering the diversity and format of the webinars. Considering the web-based community–building framework followed that of Abfalter et al [15], this study’s findings suggest that the AB-SCILS could be further improved by enhancing its strategies to promote a sense of membership among all people involved in the lives of persons with SCI as well as integrating all possible members with the common goal of fulfilling individuals’ needs by building and sharing emotional connections.

It is important to reflect on the finding that the purpose of the AB-SCILS was not clear to all attendees, suggesting that some individuals did not have their needs met by participating in the webinars. In a published article presenting 12 tips to create an effective and impactful webinar, Topor and Budson [29] suggested conducting a needs assessment with the webinar organizer and participants to learn what each group hopes to obtain from the webinar. This allows the webinar to be tailored to the needs of all stakeholders. AB-SCILS attendees may have felt that they did not have their needs met during the webinar because of learning with individuals who were outside of their usual “communities” (eg, persons with SCI vs health care providers). An article investigating whether and how the demographics of peers could influence engagement and knowledge retention suggests that social engagement matters in web-based courses [30]. Specifically, the authors found that individuals affiliated with age-similar others in a web-based course had a higher probability of course completion [30]. Consequently, the AB-SCILS could be further improved by integrating a strategy that allows for continuous consultation with community members to define the needs and priorities to build future webinars in this initiative, as well as considering a more central role of SCI peers in the delivery of AB-SCILS strategies.

The AB-SCILS had an impact on the cognitive perception of whether persons with SCI can lead meaningful, *normal* lives as the webinar content was cocreated with persons with lived experience. However, the authors also found an association between educational level and feeling *happy* or *calm* when interacting with persons with SCI as those with a postsecondary education reported these feelings more compared with those without a postsecondary education. A literature review (N=48 articles) analyzing the acceptance of employees with disabilities at work found that individuals with lower levels of education favored the segregation of individuals with disabilities [31]. Similarly, those with higher knowledge and previous experience interacting with persons with lived experience of disability had more favorable attitudes toward them. This underlines the importance of understanding more about the association between level of education and perceptions of disability as this could help tailor educational strategies to foster better community integration for people with SCI.

This study revealed that the AB-SCILS webinar platform was viewed as simple to use and easy to learn and allowed attendees to become productive quickly. A study suggested that there is no difference in the quality or acquisition of knowledge between web-based and traditional methods of learning such as self-reading, lectures, or face-to-face interaction [32]. This study’s results support this finding, reassuring that the webinar format has teaching qualities equivalent to those of other traditional methods. Therefore, implementing webinar formats to generate effective learning spaces for people with SCI and all others involved in their lives is a feasible strategy.

Finally, in terms of sustainability, it is assumed that the webinars are sustainable as they were perceived as highly usable and accessible. Furthermore, the webinars are financially sustainable as the creation of the AB-SCILS was independent from this study’s grant funding; all those who contributed to the AB-SCILS did so without receiving any additional monetary compensation (the grant funded this study). However, with changing provincial COVID-19 public health mandates, AB-SCILS contributors have had to return to their prepandemic employment demands, resulting in little to no time available to invest in the AB-SCILS. As such, the AB-SCILS has currently been put on hold, with the plan to evolve the webinars to fit within the changing climate and continue to be responsive to the needs of the community. It is essential to note that this current challenge is not because the demand for the AB-SCILS decreased. Demand has remained consistent, as evidenced by the continued viewing of past webinars on YouTube and offshoots of AB-SCILS forming through community members. Instead, this challenge has resulted from organizational changes not considered during the creation of the AB-SCILS and, therefore, presents a key learning opportunity in relation to sustainability: for the AB-SCILS to be sustainable in the long term, more organizational support and dedicated personnel are required.

### Limitations

This mixed methods study has some limitations. First, the authors did not validate or test the psychometric properties of the challenging normality survey that they created. However, this survey was developed in consultation with persons with SCI, and therefore, it was considered valid to explore this phenomenon. Second, the authors did not differentiate between time since injury in individuals with SCI and health providers’ years of experience working with SCI. This limited the authors’ ability to understand how the webinar affected people at different stages in relation to the phenomenon of SCI. Measuring time since injury and years of experience could have helped the authors further tailor the content and dissemination strategy of the AB-SCILS. Third, there was a low response rate to the survey (31/234, 13.2%). Most notably, there was a low number of health care providers who completed the surveys and, subsequently, a low number of health care providers who participated in the interviews. However, the low representation of health care providers in comparison with individuals living with SCI was expected and representative of the population who attended the AB-SCILS. Fourth, the population who participated in this study mostly identified as female. Although we do not have pop-up question data on gender, thus limiting our ability to know if this was representative of the population attending the AB-SCILS, research shows that most individuals in Canada with traumatic [33] and nontraumatic SCI [34] are male. The low representation of health care providers and male participants with SCI limited the generalizability of the survey results and the representability of this group in the interviews’ narratives. More participation from health care providers and male individuals with SCI could have provided further breadth and depth of information about the overall impact and feasibility of the AB-SCILS.

### Conclusions

The AB-SCILS, a webinar-based strategy to promote community building in SCI through the creation of a safe learning space guided by peers and clinical experts, improved participants’ knowledge of what is possible to achieve after an SCI, positively affecting their perceptions of disability. The long-term implementation of this initiative is feasible, but further considerations to increase its reach to rural and underserved areas and ensure the integration of diverse individuals, including family members and care providers, should be taken.

## Appendix

Multimedia Appendix 1 Alberta Spinal Cord Injury Community of Interactive Learning Series survey questions, corresponding surveys, and survey results (n=31).

Multimedia Appendix 2 Integrated results generated through mixed methods analysis.

## Abbreviations

AB-SCILS: Alberta Spinal Cord Injury Community of Interactive Learning Series
OR: odds ratio
REDCap: Research Electronic Data Capture
SCI: spinal cord injury

## Data Availability

The data sets generated and analyzed during this study are available from the corresponding author upon reasonable request.
