# Supplementary material for: Outcomes of Implementing a Webinar-Based Strategy to Improve Spinal Cord Injury Knowledge and Community Building: Convergent Mixed Methods Study
Source: JMIR Rehabil Assist Technol. 2023 Jun 23;10:e46575. doi: 10.2196/46575 (PMC10337322; doi:10.2196/46575)
Supplement: Multimedia Appendix 1 [file rehab_v10i1e46575_app1.docx]

**Multimedia Appendix 1.** Alberta Spinal Cord Injury Community of Interactive Learning Series (AB-SCILS) survey questions, corresponding surveys, and survey results (N=31).

| AB-SCILS survey question number, corresponding survey question, and provided answers | | | Respondents, n (%) | | Survey from which the question was adapted | |
| --- | --- | --- | --- | --- | --- | --- |
| **1: I get important needs of mine met because I am part of the AB-SCILS community.** | | | | | SOVC^a^ [15] | |
|  | Not at all | | 1 (3) | |  | |
|  | Somewhat | | 13 (42) | |  | |
|  | Mostly | | 14 (42) | |  | |
|  | Completely | | 3 (10) | |  | |
| **2: When I have a problem, I can talk about it with members of the AB-SCILS community.** | | | | | SOVC [15] | |
|  | Not at all | | 3 (10) | |  | |
|  | Somewhat | | 10 (32) | |  | |
|  | Mostly | | 11 (35) | |  | |
|  | Completely | | 7 (23) | |  | |
| **3: People in the AB-SCILS community have similar needs, priorities, and goals.** | | | | | SOVC [15] | |
|  | Not at all | | 1 (3) | |  | |
|  | Somewhat | | 3 (10) | |  | |
|  | Mostly | | 19 (61) | |  | |
|  | Completely | | 8 (26) | |  | |
| **4: I can trust people in the AB-SCILS community.** | | | | | SOVC [15] | |
|  | Not at all | | 0 (0) | |  | |
|  | Somewhat | | 3 (10) | |  | |
|  | Mostly | | 17 (55) | |  | |
|  | Completely | | 11 (35) | |  | |
| **5: Most AB-SCILS community members know me.** | | | | | SOVC [15] | |
|  | Not at all | | 8 (26) | |  | |
|  | Somewhat | | 12 (39) | |  | |
|  | Mostly | | 9 (29) | |  | |
|  | Completely | | 2 (6) | |  | |
| **6: Fitting in with the AB-SCILS community is important to me.** | | | | | SOVC [15] | |
|  | Not at all | | 2 (6) | |  | |
|  | Somewhat | | 11 (35) | |  | |
|  | Mostly | | 10 (32) | |  | |
|  | Completely | | 8 (26) | |  | |
| **7: The AB-SCILS community can influence other communities.** | | | | | SOVC [15] | |
|  | Not at all | | 0 (0) | |  | |
|  | Somewhat | | 5 (16) | |  | |
|  | Mostly | | 10 (32) | |  | |
|  | Completely | | 16 (52) | |  | |
| **8: I have influence over what the AB-SCILS community is like.** | | | | | SOVC [15] | |
|  | Not at all | | 6 (19) | |  | |
|  | Somewhat | | 18 (58) | |  | |
|  | Mostly | | 7 (23) | |  | |
|  | Completely | | 0 (0) | |  | |
| **9: If there is a problem in the AB-SCILS community, members can get it solved.** | | | | | SOVC [15] | |
|  | Not at all | | 2 (6) | |  | |
|  | Somewhat | | 12 (39) | |  | |
|  | Mostly | | 15 (48) | |  | |
|  | Completely | | 2 (6) | |  | |
| **10: The AB-SCILS community has good leaders.** | | | | | SOVC [15] | |
|  | Not at all | | 0 (0) | |  | |
|  | Somewhat | | 6 (19) | |  | |
|  | Mostly | | 10 (32) | |  | |
|  | Completely | | 15 (48) | |  | |
| **11: It is very important to me to be a part of the AB-SCILS community.** | | | | | SOVC [15] | |
|  | Not at all | | 0 (0) | |  | |
|  | Somewhat | | 7 (23) | |  | |
|  | Mostly | | 10 (32) | |  | |
|  | Completely | | 14 (45) | |  | |
| **12: I am with other community members a lot and enjoy being with them.** | | | | | SOVC [15] | |
|  | Not at all | | 3 (10) | |  | |
|  | Somewhat | | 15 (48) | |  | |
|  | Mostly | | 9 (29) | |  | |
|  | Completely | | 4 (13) | |  | |
| **13: I expect to be a part of the AB-SCILS community for a long time.** | | | | | SOVC [15] | |
|  | Not at all | | 1 (3) | |  | |
|  | Somewhat | | 2 (6) | |  | |
|  | Mostly | | 8 (26) | |  | |
|  | Completely | | 20 (65) | |  | |
| **14: I feel hopeful about the future of the AB-SCILS community.** | | | | | SOVC [15] | |
|  | Not at all | | 0 (0) | |  | |
|  | Somewhat | | 4 (13) | |  | |
|  | Mostly | | 15 (48) | |  | |
|  | Completely | | 12 (39) | |  | |
| **15: Members of the AB-SCILS community care about each other.** | | | | | SOVC [15] | |
|  | Not at all | | 0 (0) | |  | |
|  | Somewhat | | 0 (0) | |  | |
|  | Mostly | | 9 (29) | |  | |
|  | Completely | | 22 (71) | |  | |
| **16: It was simple to use the AB-SCILS webinar platform.** | | | | | TUQ^b^ [16] | |
|  | Strongly disagree | | 0 (0) | |  | |
|  | Disagree | | 0 (0) | |  | |
|  | Neither agree nor disagree | | 2 (6) | |  | |
|  | Agree | | 18 (58) | |  | |
|  | Strongly agree | | 11 (35) | |  | |
| **17: It was easy to learn to use the AB-SCILS webinar platform.** | | | | | TUQ [16] | |
|  | Strongly disagree | | 0 (0) | |  | |
|  | Disagree | | 0 (0) | |  | |
|  | Neither agree nor disagree | | 2 (6) | |  | |
|  | Agree | | 14 (45) | |  | |
|  | Strongly agree | | 15 (48) | |  | |
| **18: I believe I could become productive quickly using the AB-SCILS webinar platform.** | | | | | TUQ [16] | |
|  | Strongly disagree | | 0 (0) | |  | |
|  | Disagree | | 0 (0) | |  | |
|  | Neither agree nor disagree | | 5 (16) | |  | |
|  | Agree | | 15 (48) | |  | |
|  | Strongly agree | | 11 (35) | |  | |
| **19: Before attending this AB-SCILS session, I did not know much about _______.** | | | | | Author-created | |
|  | Strongly disagree | | 3 (10) | |  | |
|  | Disagree | | 9 (29) | |  | |
|  | Neither agree nor disagree | | 11 (35) | |  | |
|  | Agree | | 4 (13) | |  | |
|  | Strongly agree | | 3 (10) | |  | |
| **20: After attending this AB-SCILS session, I know more about _______.** | | | | | Author-created | |
|  | Strongly disagree | | 0 (0) | |  | |
|  | Disagree | | 0 (0) | |  | |
|  | Neither agree nor disagree | | 5 (16) | |  | |
|  | Agree | | 24 (77) | |  | |
|  | Strongly agree | | 2 (6) | |  | |
| **21: I think someone with a spinal cord injury can have a meaningful life.** | | | | | Author-created | |
|  | Strongly disagree | | 0 (0) | |  | |
|  | Disagree | | 0 (0) | |  | |
|  | Neither agree nor disagree | | 0 (0) | |  | |
|  | Agree | | 4 (13) | |  | |
|  | Strongly agree | | 27 (87) | |  | |
| **22: I think someone with a spinal cord injury can lead a normal life.** | | | | | Author-created | |
|  | Strongly disagree | | 0 (0) | |  | |
|  | Disagree | | 1 (3) | |  | |
|  | Neither agree nor disagree | | 2 (6) | |  | |
|  | Agree | | 11 (35) | |  | |
|  | Strongly agree | | 17 (55) | |  | |
| **23: I think that someone with a spinal cord injury can lead an independent life.** | | | | | Author-created | |
|  | Strongly disagree | | 0 (0) | |  | |
|  | Disagree | | 0 (0) | |  | |
|  | Neither agree nor disagree | | 1 (3) | |  | |
|  | Agree | | 14 (45) | |  | |
|  | Strongly agree | | 16 (52) | |  | |
| **24: I feel sorry for someone with a spinal cord injury.** | | | | | Author-created | |
|  | Never | | 4 (13) | |  | |
|  | Very rarely | | 10 (32) | |  | |
|  | Rarely | | 5 (16) | |  | |
|  | Occasionally | | 10 (32) | |  | |
|  | Frequently | | 0 (0) | |  | |
|  | Very frequently | | 2 (6) | |  | |
|  | Always | | 0 (0) | |  | |
| **25: I feel sad when I see someone with a spinal cord injury.** | | | | | Author-created | |
|  | Never | | 3 (10) | |  | |
|  | Very rarely | | 7 (23) | |  | |
|  | Rarely | | 8 (26) | |  | |
|  | Occasionally | | 10 (32) | |  | |
|  | Frequently | | 0 (0) | |  | |
|  | Very frequently | | 2 (6) | |  | |
|  | Always | | 1 (3) | |  | |
| **26: I feel happy when I see someone with a spinal cord injury.** | | | | | Author-created | |
|  | Never | | 10 (32) | |  | |
|  | Very rarely | | 3 (10) | |  | |
|  | Rarely | | 5 (16) | |  | |
|  | Occasionally | | 6 (19) | |  | |
|  | Frequently | | 0 (0) | |  | |
|  | Very frequently | | 6 (19) | |  | |
|  | Always | | 1 (3) | |  | |
| **27: I feel angry when I see someone with a spinal cord injury.** | | | | | Author-created | |
|  | Never | | 18 (58) | |  | |
|  | Very rarely | | 5 (16) | |  | |
|  | Rarely | | 3 (10) | |  | |
|  | Occasionally | | 4 (13) | |  | |
|  | Frequently | | 0 (0) | |  | |
|  | Very frequently | | 1 (3) | |  | |
|  | Always | | 0 (0) | |  | |
| **28: I feel anxious when I see someone with a spinal cord injury.** | | | | | Author-created | |
|  | Never | | 17 (55) | |  | |
|  | Very rarely | | 6 (19) | |  | |
|  | Rarely | | 2 (6) | |  | |
|  | Occasionally | | 6 (19) | |  | |
|  | Frequently | | 0 (0) | |  | |
|  | Very frequently | | 0 (0) | |  | |
|  | Always | | 0 (0) | |  | |
| **29: I feel calm when I see someone with a spinal cord injury.** | | | | | Author-created | |
|  | Never | | 2 (6) | |  | |
|  | Very rarely | | 2 (6) | |  | |
|  | Rarely | | 2 (6) | |  | |
|  | Occasionally | | 6 (19) | |  | |
|  | Frequently | | 0 (0) | |  | |
|  | Very frequently | | 10 (32) | |  | |
|  | Always | | 9 (29) | |  | |

^a^SOVC: sense of virtual community questionnaire.

^b^TUQ: Telehealth Usability Questionnaire.
