# Supplementary material for: Outcomes of Implementing a Webinar-Based Strategy to Improve Spinal Cord Injury Knowledge and Community Building: Convergent Mixed Methods Study
Source: JMIR Rehabil Assist Technol. 2023 Jun 23;10:e46575. doi: 10.2196/46575 (PMC10337322; doi:10.2196/46575)
Supplement: Multimedia Appendix 2 [file rehab_v10i1e46575_app2.docx]

**Multimedia Appendix 2** Integrated results generated through mixed methods analysis.

| Integrated category, included qualitative themes, and corresponding survey questions | | | Integrated assumptions explored | Integrated results |  |
| --- | --- | --- | --- | --- | --- |
| **Category 1: Knowledge disseminated during the webinars and its applicability** | | | | | |
|  | | **Legitimacy of knowledge** | | | |
|  |  | Question 4 | If the knowledge disseminated through the AB-SCILS^a^ was viewed as legitimate, then question 4 should show high levels of agreement. | A total of 90.3% of respondents reported “mostly” or “completely” on question 4, suggesting that the knowledge shared was viewed as legitimate. |  |
|  |  | Questions 19 and 20 | If the knowledge disseminated through the AB-SCILS was viewed as legitimate, question 19 should show moderate to high levels of agreement and question 20 should show high levels of agreement. | A total of 22.6% of respondents agreed or strongly agreed with question 19, whereas 84% agreed or strongly agreed with question 20. This suggests that attendees gained legitimate knowledge during the webinars. |  |
|  | | **Applying knowledge** | | |  |
|  |  | Question 2 | If the knowledge was applicable, then question 2 should show high levels of agreement. | A total of 58.1% of individuals with lived experience reported “mostly” or “completely” on question 2. This finding suggests that the knowledge shared during the webinars was moderately to highly applicable. |  |
|  |  | Question 3 | If the knowledge was applicable, then question 3 should show high levels of agreement. | A total of 87.1% of respondents reported “mostly” or “completely” on question 3. This suggests that the knowledge shared was applicable as most attendees could relate to it given that they had similar needs, priorities, and goals. |  |
|  |  | Question 18 | If the knowledge was applicable, then question 18 should show high levels of agreement. | A total of 83.9 % of respondents agreed or strongly agreed with question 18. This suggests that the knowledge shared was applicable as the webinars could be leveraged for productivity. |  |
|  |  | Question 21 | If the knowledge was applicable, then question 21 should show high levels of agreement. | A total of 100% of respondents agreed or strongly agreed with question 21. This suggests that the AB-SCILS provides applicable knowledge that helps attendees understand that someone with lived experience can lead a meaningful life. |  |
| **Category 2: AB-SCILS impact on community building and social connectedness** | | | | |  |
|  | | **Building community** | | |  |
|  |  | Question 2 | If the AB-SCILS helped build community, then question 2 should show high levels of agreement. | A total of 66.7% of individuals with lived experience agreed or strongly agreed with question 2. This finding suggests that community building may have been more successful among persons with lived experience as they were more likely to discuss problems with one another than individuals without lived experience. |  |
|  |  | Question 4 | If the AB-SCILS helped build community, then question 4 should show high levels of agreement. | A total of 90.3% of respondents reported “mostly” or “completely” on question 4, suggesting that the AB-SCILS helped build a trusting community. |  |
|  |  | Question 5 | If the AB-SCILS helped build community, then question 5 should show high levels of agreement. | A total of 35.6% of respondents reported “mostly” or “completely” on question 5. No subgroup analyses revealed substantial findings, suggesting that, regardless of the type of participant, gender, employment status, or educational level, most respondents did not feel that community members knew them. |  |
|  |  | Question 6 | If the AB-SCILS helped build community, then question 6 should show high levels of agreement. | A total of 58.1% of respondents reported “mostly” or “completely” on question 6. Stratifying responses by type of participant showed that 71.4% of individuals with lived experience agreed or strongly agreed with question 6 compared with 46.2% of individuals without lived experience; however, the difference was not significant. Nevertheless, this finding suggests that it is more important to an individual with lived experience to feel like they fit into the community. |  |
|  |  | Question 10 | If the AB-SCILS helped build community, then question 10 should show high levels of agreement. | A total of 80.7% of respondents reported “mostly” or “completely” on question 10. This suggests that the AB-SCILS community has good leaders that contribute to building the AB-SCILS community. |  |
|  |  | Question 12 | If the AB-SCILS helped build community, then question 12 should show high levels of agreement. | A total of 41.9% of respondents reported “mostly” or “completely” on question 12. Stratifying this result by demographics showed that 72.7% of men agreed or strongly agreed with question 12 and were almost 5 times as likely to do so (OR^b^ 4.99; *P*=.04). This finding suggests that being with other members of the community is an important part of community building for men and that women may need a different form of connection. |  |
|  |  | Question 15 | If the AB-SCILS helped build community, then question 15 should show high levels of agreement. | A total of 100% of respondents reported “mostly” or “completely” on question 15. This suggests that members of the AB-SCILS community care about each other, which is an essential component of community building. |  |
|  | | **Meeting community needs** | | |  |
|  |  | Question 1 | If the AB-SCILS met community needs (ie, there was a lack of clarity or understanding about the AB-SCILS), then question 1 should show high levels of agreement. | A total of 51.9% of respondents reported “mostly” or “completely” on question 1. In total, 66.7% of individuals with lived experience were in agreement compared with 61.5% of people without lived experience who felt like their needs were not being met. Further stratification showed that 83.3% of health care providers disagreed with question 1, suggesting that the AB-SCILS reinforces the existing SCI^c^ community but not necessarily expands it (OR 0.011; *P*=.06). |  |
| **Category 3: AB-SCILS impact on SCI perceptions of normality and disability** | | | | |  |
|  | | **Challenging normality** | | |  |
|  |  | Question 21 | If the AB-SCILS helped challenge ideas of what “normal” life with SCI looks like, then question 21 should show high levels of agreement. | A total of 100% of respondents agreed or strongly agreed with question 21. This suggests that the AB-SCILS helps attendees understand that someone with lived experience can lead a meaningful life. |  |
|  |  | Question 22 | If the AB-SCILS helped challenge ideas of what “normal” life with SCI looks like, then question 22 should show high levels of agreement. | A total of 90.3% of respondents agreed or strongly agreed with question 22. This suggests that the AB-SCILS helps attendees understand that someone with lived experience can lead a normal life. |  |
|  |  | Question 23 | If the AB-SCILS helped challenge ideas of what “normal” life with SCI looks like, then question 23 should show high levels of agreement. | A total of 93.8% of respondents agreed or strongly agreed with question 23. This suggests that the AB-SCILS helps attendees understand that someone with lived experience can lead an independent life. |  |
|  |  | Question 24 | If the AB-SCILS helped challenge ideas of what “normal” life with SCI looks like, then question 24 should show low levels of agreement. | A total of 64.71% of respondents disagreed or strongly disagreed with question 24. Stratifying results by demographics did not reveal any significant findings, suggesting that a variety of attendees held this view of feeling sorry for someone with an SCI. |  |
|  |  | Question 25 | If the AB-SCILS helped challenge ideas of what “normal” life with SCI looks like, then question 25 should show low levels of agreement. | A total of 61.3% of respondents reported “never,” “very rarely,” or “rarely” on question 25. In total, 63% of men agreed or strongly agreed with question 25, which suggests that the AB-SCILS may need to consider gender differences when it is used as a platform to challenge notions of what normal life looks like with SCI in the future. |  |
|  |  | Question 26 | If the AB-SCILS helped challenge ideas of what “normal” life with SCI looks like, then question 26 should show high levels of agreement. | A total of 42% of respondents reported “occasionally,” “frequently,” “very frequently,” or “always” on question 26. Attendees with a postsecondary education were almost 13 times more likely to report feeling happy when seeing someone with an SCI (OR 12.6; *P*=.03). This suggests that higher education may make individuals more receptive and open to challenging perceptions of what normal life with SCI looks like. |  |
|  |  | Question 27 | If the AB-SCILS helped challenge ideas of what “normal” life with SCI looks like, then question 27 should show low levels of agreement. | A total of 83.9% of respondents reported “never,” “very rarely,” or “rarely” on question 27, suggesting that the AB-SCILS challenged ideas of normality. |  |
|  |  | Question 28 | If the AB-SCILS helped challenge ideas of what “normal” life with SCI looks like, then question 28 should show low levels of agreement. | A total of 80.8% of respondents reported “never,” “very rarely,” or “rarely” on question 27, suggesting that the AB-SCILS challenged ideas of normality. |  |
|  |  | Question 29 | If the AB-SCILS helped challenge ideas of what “normal” life with SCI looks like, then question 29 should show high levels of agreement. | A total of 80.7% of respondents reported “occasionally,” “frequently,” “very frequently,” or “always” on question 29. Stratification by educational level revealed that those with a postsecondary education were 11 times more likely to report feeling calm compared with those without a postsecondary education (*P*=.01). This suggests that higher education may make individuals more receptive and open to challenging perceptions of what normal life with SCI looks like. |  |
| **Category 4: AB-SCILS usability** | | | | |  |
|  | | **Webinar platform usability** | | |  |
|  |  | Question 16 | If the webinar platform was perceived as highly usable, question 16 should show high levels of agreement. | A total of 93.6% of respondents agreed or strongly agreed with question 16, suggesting that the simplicity of the platform contributed to perceptions of its usability. |  |
|  |  | Question 17 | If the webinar platform was perceived as highly usable, question 17 should show high levels of agreement. | A total of 90.6% of respondents agreed or strongly agreed with question 17, suggesting that ease of learning how to use the platform contributed to perceptions of its usability. |  |
|  |  | Question 18 | If the webinar platform was perceived as highly usable, question 18 should show high levels of agreement. | A total of 83.9% of respondents agreed or strongly agreed with question 18, suggesting that the ability to become productive quickly using the platform contributed to perceptions of its usability. |  |

^a^AB-SCILS: Alberta Spinal Cord Injury Community of Interactive Learning Series.

^b^OR: odds ratio.

^c^SCI: spinal cord injury.
